# Coculture of *Collinsella aerofaciens* and *Bacteroides thetaiotaomicron* under bile acid stress reveals vitamin B6 exchange

**DOI:** 10.64898/2026.08.03.742426

**Published:** 2026-08-03

**Authors:** Yan Wang, Sven-Bastiaan Haange, Andy Mercado Gamarra, Martin Leníček, Deborah Maier, Robin Plail, Hendrik Seidl, Christoph Kaleta, Ulrike Rolle-Kampczyk, Nico Jehmlich, Martin von Bergen

## Abstract

Although bile acid-mediated microbiome-host interactions are known to shape gut microbial composition and function, the mechanisms by which bile acid stress influences microbial metabolic interactions remain poorly understood. Here, we investigated the interaction between two key gut microbes, *Bacteroides thetaiotaomicron* and *Collinsella aerofaciens* , under deoxycholic acid (DCA) stress. In anaerobic coculture, *C. aerofaciens* mitigated the inhibitory effects of DCA on *B. thetaiotaomicron* , primarily through DCA uptake from the medium, as confirmed by DCA quantification. Proteomic analysis showed that DCA broadly disrupted amino acid and vitamin metabolism, particularly in *B. thetaiotaomicron* . In contrast, coculture promoted widespread metabolic activation in *C. aerofaciens* , including enhanced vitamin B6 metabolism and increased production of citrulline and ornithine. These findings suggest that metabolic cooperation enhances resistance to bile acid stress and may contribute to gut microbiome resilience, with potential relevance to liver- and bile acid-related disorders.

## Full Text Availability

The license terms selected by the author(s) for this preprint version do not permit archiving in PMC. The full text is available from the preprint server.

